# Pediatric spinal ependymomas: Long‐term surgical outcomes in a cohort of 61 cases

**DOI:** 10.1002/ped4.70045

**Published:** 2026-02-09

**Authors:** Liang Zhang, Xingyu Liu, Bo Han, Wenqing Jia

**Affiliations:** ^1^ Department of Neurosurgery National Cancer Center/National Clinical Research Center for Cancer/Cancer Hospital Chinese Academy of Medical Sciences and Peking Union Medical College Beijing China; ^2^ Department of Neurosurgery Beijing Tiantan Hospital Capital Medical University Beijing China

**Keywords:** Ependymomas, Myxopapillary ependymoma, Pediatric, Recurrence, Spinal, Subependymoma

## Abstract

**Importance:**

Spinal ependymomas are rare in the pediatric population, with limited evidence of long‐term outcomes and predictors of recurrence.

**Objective:**

To analyze clinic‐radiological features, therapeutic methods, and long‐term outcomes in a pediatric cohort.

**Methods:**

We retrospectively reviewed patients ≤18 years who underwent surgical resection for spinal ependymomas between January 2012 and July 2024. Progression‐free survival (PFS) was estimated using Kaplan‐Meier analysis, and predictors of recurrence were analyzed using the Cox proportional hazards method.

**Results:**

Among 61 children (age 13.7 ± 3.7 years), spinal ependymoma (EPN) (WHO grade 2) was the most common subtype (*n* = 36, 59.0%), followed by myxopapillary ependymoma (MPE) (*n* = 13, 21.3%), EPN (WHO grade 3) (*n* = 11, 18.1%) and subependymoma (SE) (*n* = 1, 1.6%). Gross‐total resection (GTR) was achieved in 38 patients (62.3%). Over a follow‐up of 42.8 ± 34.9 months, 19 patients (31.1%) experienced tumor recurrence. Functional improvement was observed in 38 children (62.3%). The 5‐ and 10‐year progression‐free survival (PFS) rates were 73.4% and 59.6%, respectively. In grade 2 EPN, subtotal resection (STR) followed by radiotherapy yielded significantly better 5‐ and 10‐year PFS than STR alone (100% vs. 42.9%; 66.7% vs. 21.4%, respectively). Multivariable analysis identified extent of resection (*P* = 0.015), MPE subtype (*P* = 0.014), and Ki‐67 ≥8% (*P* = 0.001) as independent predictors of recurrence.

**Interpretation:**

GTR remains the best treatment modality for pediatric patients with spinal ependymomas and has a favorable prognosis. Tumor recurrence is common and is related to the Ki‐67 index, histological subtype, and the extent of resection.

## Graphical abstract

Spinal ependymomas are rare in children, with limited long‐term outcome data. In this retrospective study of 61 pediatric patients undergoing surgical resection, gross total resection was achieved in 62.3% and was associated with favorable functional improvement. Recurrence was observed in 31.1% of cases. Independent predictors of recurrence included extent of resection, myxopapillary subtype, and Ki‐67 index ≥8%.

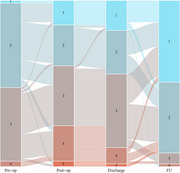

## INTRODUCTION

Spinal ependymomas are neuroepithelial tumors of the central nervous system (CNS) that typically manifest during the 3^rd^–4^th^ decades of life, with no significant sex predilection.^1^, ^2^, ^3^ While spinal ependymomas account for approximately 75% of all ependymomas in adults, they are exceedingly rare in the pediatric population, representing only 8%–13% of childhood ependymomas.^4^, ^5^ The 2021 WHO classification of CNS tumors distinguishes four molecular and histological subtypes of spinal ependymomas: spinal subependymoma (SE), spinal myxopapillary ependymoma (MPE), spinal ependymoma (EPN), and *MYCN*‐amplified spinal ependymoma (EPN‐MYCN).^6^

Compared to their adult counterparts, pediatric patients with spinal ependymomas exhibit distinct clinicopathological features, including presentation, biological behavior, and survival outcomes.^4^, ^5^, ^7^, ^8^ However, existing literature was mainly reported conjointly with adults, and pediatric case series were limited,^9^, ^10^, ^11^, ^12^, ^13^, ^14^, ^15^, ^16^ resulting in critical knowledge gaps regarding clinical‐radiological manifestations, management protocols, and prognosis in children. To address these problems, we present a large, single‐center, retrospective analysis of pediatric patients with spinal ependymomas that systematically delineates their clinical‐radiological features and surgical outcomes.

## METHODS

### Ethical approval

The study was approved by the Institutional Review Board (IRB) of Beijing Tiantan Hospital, Capital Medical University (No. 25/411‐5357 and KY2022‐089‐02). Written informed consent was obtained from patients’ legal guardians. All the procedures conformed to the ethical standards of the Declaration of Helsinki.

### Study population

Pediatric patients (aged ≤18 years) who underwent surgical resection for spinal ependymomas at our institution between January 2012 and July 2024 were enrolled. Children diagnosed with NF2‐related (neurofibromatosis type 2) schwannomatosis due to clinical characteristics and/or a germline mutation in the *NF2* gene and ependymomas originating from the brain and spreading to the spinal cord were excluded from this study. The following data were extracted from electronic medical records: baseline demographics, preoperative magnetic resonance imaging (MRI) characteristics, operative details, neuropathology reports, adjuvant radiotherapy (RT) and/or chemotherapy, early postoperative course, and follow‐up information.

### Function measurements

Neurological status was graded pre‐ and postoperatively using the modified McCormick Scale (mMCS) (Table S1).^17^ Functional outcomes were evaluated preoperatively, immediately after surgery, at discharge, and at the last follow‐up in July 2025.

### MRI evaluation

Tumor size was defined as the largest enhancing diameter (transverse, anteroposterior, or craniocaudal) on the contrast‐enhanced T1‐weighted sequences. The tumor volume was calculated as follows: (transverse × anteroposterior × craniocaudal dimensions)/2. The extent of resection (EOR) was assessed based on the postoperative MRI obtained within 72 h. Gross total resection (GTR) was defined as the complete removal of all contrast‐enhancing tumors, including metastatic foci. Subtotal resection (STR) was defined as any detectable residual‐enhancing tumor.

### Surgical details

Following a comprehensive preoperative assessment, all patients were deemed suitable for microsurgical resection. The standard posterior approach was used in this study. After a laminectomy, midline myelotomy was performed along the posterior median sulcus to expose the solid component of the intramedullary tumor. Lesions typically appear as reddish‐grey, soft, and moderately vascular lesions. The tumor‐cord interface was meticulously delineated with a microdissector and forceps, proceeding in a craniocaudal direction from one tumor pole to the other. In cases involving the lumbar or sacral regions, tumors frequently adhered to the cauda equina; therefore, stepwise debulking with nerve root‐sparing dissection was performed.^18^ Leptomeningeal dissemination parts in two patients were not resected.

### Outcomes

RT and/or chemotherapy were prescribed according to the EOR and multidisciplinary tumor board review on an individualized basis. Recurrence was defined radiologically as either local regrowth or a *de novo* lesion distant from the original site. Progression‐free survival (PFS) was calculated from the date of surgery to the first documented relapse or radiological progression.

### Statistical analysis

Continuous variables were expressed as mean ± standard deviation (SD) for normally distributed data or median and interquartile range (IQR) for abnormally distributed data. Categorical variables are expressed as frequencies and percentages. Between‐group differences were analyzed using the Student's *t* test or the Mann‐Whitney *U* test for continuous variables and the Chi‐square test for categorical variables. Kaplan‐Meier curves and Cox proportional hazards regression were used to assess the association between baseline variables and PFS. Independent predictors of progression were identified using multivariable Cox models. All analyses were performed using IBM SPSS Statistics for Windows (version 25.0; IBM Corp., Armonk, NY, USA) and R software (version 4.4.1). A two‐tailed *P*‐value < 0.05 was considered statistically significant.

## RESULTS

### Baseline characteristics

Sixty‐one consecutive pediatric patients (age: 13.7 ± 3.7 years; range: 7.0–18.0 years) were enrolled (Figure S1). The male‐to‐female ratio was 2.1:1. The predominant presenting symptoms were pain (*n* = 45, 73.8%), limb weakness (*n* = 36, 59.0%), sensory disturbance (*n* = 19, 31.1%), and sphincter dysfunction (*n* = 17, 27.9%). Two patients (3.3%) exhibited extremity muscle atrophy. The median duration of symptoms was 10.0 (IQR: 3.0–16.0) months. Preoperative mMCS was ≤2 in most cases (*n* = 32, 52.5%). Most baseline characteristics, including sex, preoperative symptoms, duration of symptoms, and mMCS, did not differ significantly between the younger (≤13 years) and older (>13 years) age groups (Table 1).

**TABLE 1:** Comparison of demographic, clinical, and outcome characteristics between younger and older patient groups

Variable	Total (*n* = 61)	Young group (age ≤13 years) (*n* = 26)	Old group (age >13 years) (*n* = 35)	*P‐*value
Age (years)	13.7 ± 3.7	10.0 ± 2.2	16.5 ± 1.5	<0.001
Sex				0.400
Male	41 (67.2)	19 (73.1)	22 (62.9)	
Female	20 (32.8)	7 (26.9)	13 (37.1)	
Symptoms				
Pain	45 (73.8)	21 (80.8)	24 (68.6)	0.284
Motor	36 (59.0)	16 (61.5)	20 (57.1)	0.730
Sensory^†^	19 (31.1)	5 (19.2)	14 (40.0)	0.083
Sphincter disorders	17 (27.9)	8 (30.8)	9 (25.7)	0.663
Duration of symptoms (months)	10.0 (3.0–16.0)	6.0 (1.6–12.8)	12.0 (3.8–21.0)	0.270
Pre‐operative mMCS				0.884
1	1 (1.6)	0 (0)	1 (2.9)	
2	31 (50.8)	12 (46.2)	19 (54.2)	
3	27 (44.3)	13 (50.0)	14 (40.0)	
4	2 (3.3)	1 (3.8)	1 (2.9)	
Location				
Lumbar	33 (54.1)	15 (57.7)	18 (51.4)	0.627
Thoracic	31 (50.8)	9 (34.6)	22 (62.9)	0.029
Sacral	18 (29.5)	8 (30.8)	10 (28.6)	0.852
Cervical	18 (29.5)	4 (15.4)	14 (40.0)	0.037
Maximum size (cm)	7.1 ± 4.6	6.7 ± 5.1	7.4 ± 4.3	0.601
Maximum volume (cm^3^)	7.5 (3.8–11.3)	6.8 (4.0–9.2)	8.3 (3.2–12.1)	0.385
Maximum size ≥6 cm	29 (47.5)	14 (53.8)	15 (42.9)	0.395
Associated cyst/syrinx	24 (39.3)	10 (38.5)	14 (40.0)	0.903
Site				0.413
Intramedullary	33 (54.1)	13 (50.0)	20 (57.1)	
Extramedullary	26 (42.6)	13 (50.0)	13 (37.2)	
Intra‐extramedullary	2 (3.3)	0 (0)	2 (5.7)	
Radiotherapy	9 (14.8)	5 (19.2)	4 (11.4)	0.628
EOR				0.916
GTR	38 (62.3)	16 (61.5)	22 (62.9)	
STR	23 (37.7)	10 (38.5)	13 (37.1)	
Ki‐67 (%)	8.0 (5.0–10.5)	9.0 (5.0–16.3)	6.5 (4.8–10.0)	0.268
Ki‐67 ≥8%	30 (49.2)	13 (50.0)	17 (48.6)	0.912
WHO grade				0.318
1	1 (1.6)	1 (3.8)	0 (0)	
2	49 (80.3)	19 (73.1)	30 (85.7)	
3	11 (18.1)	6 (23.1)	5 (14.3)	
Pathology				0.575
SE	1 (1.6)	1 (3.8)	0 (0)	
MPE	13 (21.3)	5 (19.2)	8 (22.9)	
EPN (WHO grade 2)	36 (59.0)	14 (53.9)	22 (62.9)	
EPN (WHO grade 3)	11 (18.1)	6 (23.1)	5 (14.2)	
Recurrence	19 (31.1)	9 (34.6)	10 (28.6)	0.614
Recurrence time (months)	30.0 (22.5–48.0)	30.0 (21.5–48.3)	30.0 (24.0–48.0)	0.858
Outcomes				0.001
Improved	38 (62.3)	22 (84.6)	16 (45.7)	
Same	19 (31.1)	2 (7.7)	17 (48.6)	
Worse	4 (6.6)	2 (7.7)	2 (5.7)	

Data were shown as *n* (%), mean ± standard deviation, or median (interquartile range).

Abbreviations: EOR, extent of resection; EPN, spinal ependymoma; GTR, gross total resection; mMCS, modified McCormick scale; MPE, spinal myxopapillary ependymoma; SE, spinal subependymoma; STR, subtotal resection.

^†^ Sensory symptoms included sensory loss and numbness.

### MRI features

Spinal MRI revealed lesions predominantly involving the lumbar (*n* = 33, 54.1%) and thoracic (*n* = 31, 50.8%) segments. Eight patients (13.1%) harbored multifocal lesions, and two (3.3%) had leptomeningeal dissemination at diagnosis. Maximum lesion size was 7.1 ± 4.6 (range: 1.5–24.3) cm and the maximum volume was 7.5 (IQR: 3.8–11.3) cm^3^. Tumors were intramedullary in 33 patients (54.1%), extramedullary in 26 (42.6%), and intra/extramedullary in 2 (3.3%). A significantly higher proportion of older children exhibited a cervical or thoracic location (*P =* 0.037 and 0.029, respectively) (Table 1). A solid tumor component without cystic changes was present in 37 cases (60.7%), whereas 24 (39.3%) exhibited cystic degeneration. On T1‐ and T2‐weighted sequences, the solid component was predominantly iso‐ or mixed‐intensity and showed a heterogeneous enhancement. Syringomyelia coexisted in 20 patients (32.8%), and intratumoral hemorrhage was identified in 12 (19.7%). Tumor extension across ≥4 vertebral levels was observed in 40 patients (65.6%).

### Surgical treatment

A clear plane was absent in 23 cases (37.7%) due to infiltrative growth into the spinal cord or dense adhesion to the cauda equina. Therefore, STR was performed instead of total resection. GTR was achieved in the remaining 38 patients (62.3%). The median intra‐operative blood loss was 100 mL (IQR: 100–200 mL), and one child required a blood transfusion.

Histopathology demonstrated SE in one case (1.6%), MPE in 13 cases (21.3%), WHO grade 2 EPN in 36 cases (59.0%), and WHO grade 3 EPN in 11 cases (18.1%). EPN‐MYCN variants were not identified in this cohort. The median MIB‐1 index was 8.0% (IQR: 5.0%–10.5%). Intratumoral hemorrhage was observed in 12 specimens (19.7%), and degenerative changes, including necrosis, were present in seven specimens (11.5%).

Four children (6.6%) had a fever that was assumed to be from meningitis; all responded to lumbar cerebrospinal fluid drainage and antibiotics and were discharged fully recovered. The duration of hospitalization after surgery for these patients was 14.3 ± 3.0 days (range: 11.0–18.0 days). Worsening of lower‐limb weakness occurred in 19 patients (31.1%); five of them returned to preoperative baseline, and three achieved partial recovery before discharge.

### Adjuvant therapy

Adjuvant therapy was administered to 12 patients (19.7%). RT was administered to nine patients (14.8%); six received localized RT at a median cumulative dose of 50.0 (range: 48.6–60.0) Gy, and three underwent craniospinal irradiation with a median dose of 35.2 (range: 24.0–40.0) Gy and a boost dose of 48.2 (range: 40.0–55.0) Gy. The indications for RT included residual tumor and/or leptomeningeal dissemination (*n* = 8) and anaplastic histology (*n* = 3). Chemotherapy (nimustine, bevacizumab, or etoposide) was administered to three patients (4.9%) due to tumor recurrence. Conventional fractionated irradiation (single daily dose of 1.6–1.8 Gy) was delivered to six patients (M+: *n* = 4), while hyperfractionated RT (2×1.0 Gy) was used to three patients (M+: *n* = 2).

### Outcomes

During a follow‐up of 84.0 ± 48.5 months (range: 12.0–166.0 months), tumor recurrence was documented in 19 patients (31.1%); no deaths occurred. The time to recurrence was 30.0 (IQR: 22.5–48.0) months after surgery. Fourteen patients developed local recurrence, and five exhibited leptomeningeal dissemination. Seventeen patients underwent repeated surgery, and two asymptomatic patients with local recurrence were elected for surveillance. MPE subtype had the highest recurrence rate (7/13, 53.8%), followed by WHO grade 2 EPN (9/36, 25.0%) and WHO grade 3 EPN (3/11, 27.2%).

**FIGURE 1:**
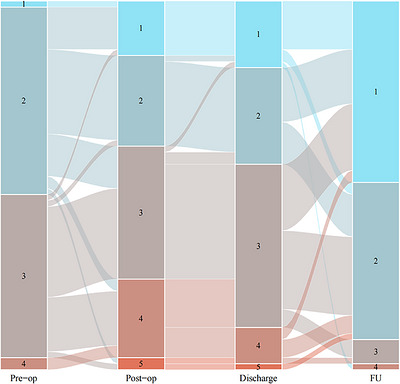
Sankey diagram illustrating changes in the mMCS throughout the operative timeline: pre‐operative (Pre‐op), immediate post‐operative (Post‐op), discharge, and follow‐up (FU). The proportions of patients with mMCS grades 1–5 were shown in different colors. mMCS, modified McCormick scale.

mMCS at last follow‐up was grade 1 in 30 (49.2%), grade 2 in 26 (42.6%), grade 3 in 4 (6.6%), and grade 4 in 1 (1.6%). Overall, 38 children (62.3%) showed functional improvement, with a significantly higher proportion observed in younger patients (Table 1 and Figure 1).

**FIGURE 2:**
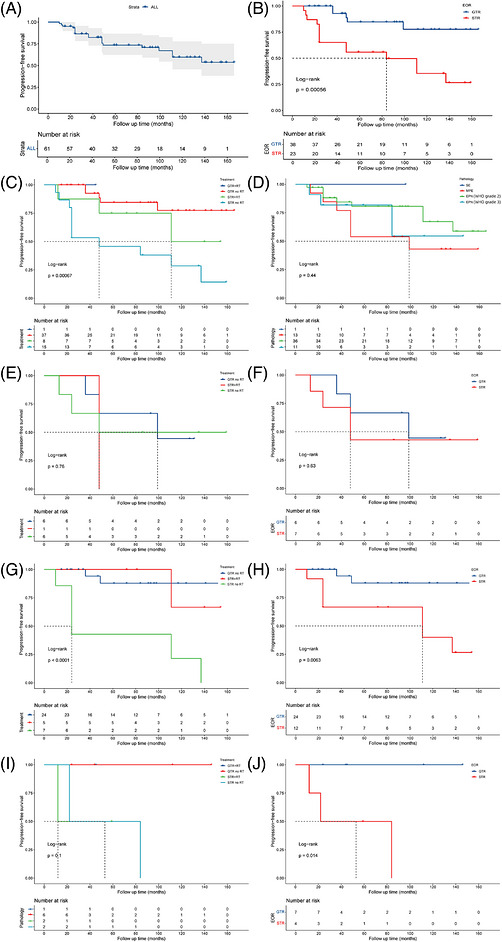
Kaplan‐Meier curves of Progression‐Free Survival (PFS). (A) PFS for the entire cohort. (B, C) PFS stratified by EOR and adjuvant treatment modality. (D) PFS stratified by histological subtype. (E, F) PFS in patients with MPE: neither treatment modality nor EOR significantly influenced PFS. (G, H) PFS in patients with Grade 2 EPN subtype: STR + RT was associated with substantially prolonged PFS compared to STR alone, and GTR further improved PFS relative to STR. (I, J) PFS in patients with Grade 3 EPN, analyzed by treatment modality and EOR. EOR, extent of resection; GTR, gross total resection; STR, subtotal resection; SE, spinal subependymoma; MPE, spinal myxopapillary ependymoma; EPN, spinal ependymoma; RT, radiotherapy.

For the entire cohort, 5‐ and 10‐year PFS were 73.4% and 59.6%, respectively (Figure 2A). GTR and postoperative RT conferred a significant PFS benefit (Figure 2B, C), whereas the histological subtype did not (Figure 2D). Among 13 patients with MPE, those who achieved GTR (*n* = 6) demonstrated 5‐ and 10‐year PFS of 66.7% and 44.4%, respectively; those who underwent STR (*n* = 7) had PFS of 42.9% throughout follow‐up (Figure 2E, F). Within Grade 2 EPN, all 24 patients who underwent GTR maintained 87.8% PFS at both 5 and 10 years (Figure 2H). Patients with STR followed by RT (*n* = 5) achieved 5‐ and 10‐year PFS of 100% and 66.7%, whereas those with STR without RT (*n* = 7) had markedly inferior PFS of 42.9% and 21.4%, respectively (Figure 2G, H). For Grade 3 EPN, seven patients who underwent GTR had 100% PFS at 5 and 10 years. Among four patients with STR, 5‐ and 10‐year PFS were 50% and 0%, respectively (Figure 2I, J).

Cox regression analysis was performed to identify predictors of recurrence. Shorter symptom duration, lumbosacral location, extramedullary extension, STR, and high Ki‐67 index (≥8%) were all significantly associated with an increased risk of recurrence (Table 2). In multivariate analysis, MPE subtype (HR: 3.922, 95% CI: 1.312–11.722, *P* = 0.014) and high Ki‐67 index (HR: 6.585, 95% CI: 2.129–20.367, *P* = 0.001) were predictors of recurrence, and the GTR reduced the risk of tumor recurrence (HR: 0.259, 95% CI: 0.088–0.766, *P* = 0.015) (Table 3).

**TABLE 2:** Comparison of baseline characteristics between patients who did and didn't experience recurrence

Variable	Recurrence (*n* = 19)	Stable (*n* = 42)	*P‐*value
Age (years)	13.4 ± 3.9	13.9 ± 3.7	0.608
Sex			0.469
Male	14 (73.7)	27 (64.3)	
Female	5 (26.3)	15 (35.7)	
Duration of symptoms (months)	6.0 (1.5–12.0)	12.0 (3.0–22.0)	0.010
Pre‐operative mMCS ≤2	13 (68.4)	19 (45.2)	0.093
Location			
Lumbar	15 (78.9)	18 (42.9)	0.009
Thoracic	8 (42.1)	23 (54.8)	0.360
Sacral	12 (63.2)	6 (14.3)	<0.001
Cervical	2 (10.5)	16 (38.1)	0.029
Maximum size (cm)	7.3 ± 5.3	6.6 ± 4.2	0.527
Maximum volume (cm^3^)	8.5 (4.4–10.4)	6.8 (3.8–11.3)	0.772
Maximum size ≥6 cm	10 (52.6)	19 (45.2)	0.592
Associated cyst/syrinx	5 (26.3)	19 (45.2)	0.161
Site			0.003
Intramedullary	5 (26.3)	28 (66.7)	
Extramedullary	14 (73.7)	12 (28.5)	
Intra‐extramedullary	0 (0)	2 (4.8)	
Radiotherapy	3 (15.8)	6 (14.3)	1.000
EOR			<0.001
GTR	5 (26.3)	33 (78.6)	
STR	14 (73.7)	9 (21.4)	
Ki‐67 (%)	9.0 (7.3–10.0)	5.0 (4.8–12.8)	0.575
Ki‐67 ≥8%	15 (78.9)	15 (35.7)	0.002
WHO grade			1.000
1	0 (0)	1 (2.4)	
2	16 (84.2)	33 (78.6)	
3	3 (15.8)	8 (19.0)	
Pathology			0.236
SE	0 (0)	1 (2.4)	
MPE	7 (36.8)	6 (14.3)	
EPN (WHO grade 2)	9 (47.4)	27 (64.3)	
EPN (WHO grade 3)	3 (15.8)	8 (19.0)	
Follow‐up (months)	79.9 ± 47.7	85.8 ± 49.2	0.661

Data were shown as *n* (%), mean ± standard deviation, or median (interquartile range).

Abbreviations: EOR, extent of resection; EPN, spinal ependymoma; GTR, gross total resection; mMCS, modified McCormick scale; MPE, spinal myxopapillary ependymoma; SE, spinal subependymoma; STR, subtotal resection.

**TABLE 3:** Univariate and multivariate Cox regression analysis of recurrence risk factors

Variable	Univariate analysis		Multivariate analysis	
	HR (95% CI)	*P*‐value	HR (95% CI)	*P*‐value
Age (years)	0.978 (0.872–1.096)^†^	0.697		
Female	0.782 (0.281–2.177)	0.638		
Duration of symptom (months)	0.948 (0.894–1.005)^†^	0.073		
Intramedullary	0.273 (0.098–0.759)	0.013	0.955 (0.114–8.018)	0.966
Lumbar location	0.303 (0.100–0.916)	0.034	1.541 (0.267–8.899)	0.629
Sacral location	0.166 (0.065–0.424)	<0.001	0.437 (0.084–2.282)	0.326
MPE	2.724 (1.090–6.804)	0.032	3.922 (1.312–11.722)	0.014
EPN	2.125 (0.848–5.323)	0.108		
GTR	0.217 (0.082–0.571)	0.002	0.259 (0.088–0.766)	0.015
WHO grade	0.832 (0.227–3.046)	0.782		
Ki‐67 ≥8%	5.896 (2.277–15.264)	<0.001	6.585 (2.129–20.367)	0.001

Abbreviations: CI, confidence interval; EPN, spinal ependymoma; GTR, gross total resection; HR, hazard ratio; MPE, spinal myxopapillary ependymoma.

^†^ Hazard ratio represents the increase in risk per 1 unit increase in the variable.

## DISCUSSION

This is one of the largest single‐center studies focusing exclusively on spinal ependymomas in the pediatric population. We found that tumor recurrence was common and was related to the Ki‐67 index, MPE subtype, and EOR. Surgery is the principal treatment and can benefit children even in relapsed cases. The surgical outcomes were usually favorable, with the most improved neurological function.

The cohort's demographic profile (mean age 13.7 years; male preponderance) was inconsistent with previous reports.^5^, ^7^, ^10^, ^11^, ^12^ Leg pain, low back pain, and weakness were the dominant presenting features, underscoring the propensity of these tumors to usually affect the lower thoracic and lumbosacral regions and disturb the nerve root. The absence of age‐related differences in baseline features, including histological grade, suggests that the biological behavior of pediatric spinal ependymomas is largely conserved across the 7–18 years spectrum.

Surgical resection is the mainstay treatment for spinal ependymomas because it can protect the already injured spinal cord or nerve roots and achieve favorable long‐term outcomes. As confirmed in this study, GTR can provide superior long‐term PFS compared with STR. However, resection of intramedullary lesions and MPE are considered high‐risk procedures. As was seen in this cohort, early neurological deterioration, worsening paraparesis common and affected 31.1% of patients, yet 42.1% of these children recovered fully or partially before discharge. This transient decline likely reflects the surgical manipulation of an already compressed spinal cord or disturbances of the nerve roots, rather than permanent injury. Importantly, younger patients had better functional outcomes than older patients, corroborating the greater neuroplastic potential of the spinal cord.^19^

Histopathological distribution was similar to that observed in adults and pediatric ependymomas.^1^, ^20^ The prevalence of classic WHO grade 2 EPN (59.0%) and the scarcity of WHO grade 3 EPN (18.1%) are consistent with other pediatric spinal ependymoma cohorts.^11^, ^15^, ^20^, ^21^ MPE—traditionally considered a conus‐filum tumor of young adults—accounted for 21.3% of our series, and no *MYCN*‐amplified subtypes were identified, as *MYCN* expression was assessed in all cases following the publication of the 2021 WHO classification of CNS tumors. This may be because of the low prevalence of this subtype.^10^, ^22^

In this cohort, we found that MPE had a higher recurrence rate, and GTR conferred superior long‐term control within this subgroup, although this was not statistically significant. Previous report supports the benefit of adjuvant RT in the treatment of MPE, with a recurrence rate of 16.7% (2/12) following STR and RT compared to 65% (13/20) after GTR alone.^20^ In a large study including 32 young patients (≤22 years) with MPE, Engertsberger et al.^10^ observed an improved PFS with adjuvant RT compared to a wait‐and‐see approach. One study including 12 disseminated MPE pediatric patients found that the 5‐year PFS was 92%, with only out‐of‐field recurrence and no in‐field recurrence.^23^ Another study including 15 pediatric cases of MPE showed the benefit of RT delivered to patients with GTR, but no such advantage in patients who received STR.^12^ These findings must be interpreted with caution as they are based on only small case numbers. The 5‐ and 10‐year PFS rates were higher in the RT group in our cohort, but the difference was not statistically significant. The limited amount of our data also precludes drawing conclusions regarding the efficacy of RT in the treatment of pediatric ependymomas. Considering the relatively small series of this rare entity, the role of adjuvant RT still needs to be verified in further studies and long‐term follow‐ups.

In this study, children with spinal ependymomas had a favorable prognosis, as no deaths were recorded and 62.3% improved their function in a mean follow‐up of 84 months in our cohort. Nonetheless, 31% developed progression at a median follow‐up of 43 months. Multivariate analysis identified MPE subtype, a high Ki‐67 index (≥8%), and the EOR as the significant factors of recurrence, findings aligned with adult series and other pediatric reports establishing EOR as the principal determinant of outcome.^3^, ^10^, ^20^, ^21^, ^24^, ^25^ Further studies to explore the molecular aspects of pediatric ependymomas need to be conducted to identify prognostic factors and therapeutic targets.

This study has several limitations. First, the retrospective, single‐center nature of the study may have led to a selection bias. Second, the rarity and limited number of MPE and grade 3 EPN preclude definitive conclusions for these subgroups and hampers evaluation of RT's long‐term impact of RT. Third, only a small number (eight of 23) of patients received RT after STR in this cohort, and the survival advantage of RT requires further study.

In summary, safe and maximal surgical resection remains the mainstay treatment for children with spinal ependymomas, yielding generally favorable outcomes with neurological improvement in most patients. Tumor recurrence is common and related to the Ki‐67 index, histological subtype, and EOR. Future multicenter trials incorporating molecular stratification and patient‐reported outcome measures are essential to refine risk‐adapted management strategies for these rare tumors.

## CONFLICT OF INTEREST

The authors declare no conflict of interest.

## Supporting information



Supporting Information
