# Supplementary material for: Pediatric spinal ependymomas: Long‐term surgical outcomes in a cohort of 61 cases
Source: Pediatr Investig. 2026 Feb 9;10(3):240–8. doi: 10.1002/ped4.70045 (PMC13291426; doi:10.1002/ped4.70045)
Supplement: Supplementary file 1 — Supporting Information [file PED4-10-240-s001.pdf]

Supplementary Material for

Pediatric spinal ependymomas: Long-term surgical outcomes in a cohort of 61 cases

Liang Zhang, Xingyu Liu, Bo Han, Wenqing Jia

Table S1. The modified McCormick scale (MMCS) for functional classification of pediatric patients with spinal ependymomas

Grade	Definition
1	Neurologically intact, ambulates normally, may have minimal dysesthesia
2	Mild motor or sensory deficit, maintains functional independence
3	Moderate deficit, limitation of function, independent with external aid
4	Severe motor or sensory deficit, dependent with external aid
5	Paraplegia or quadriplegia

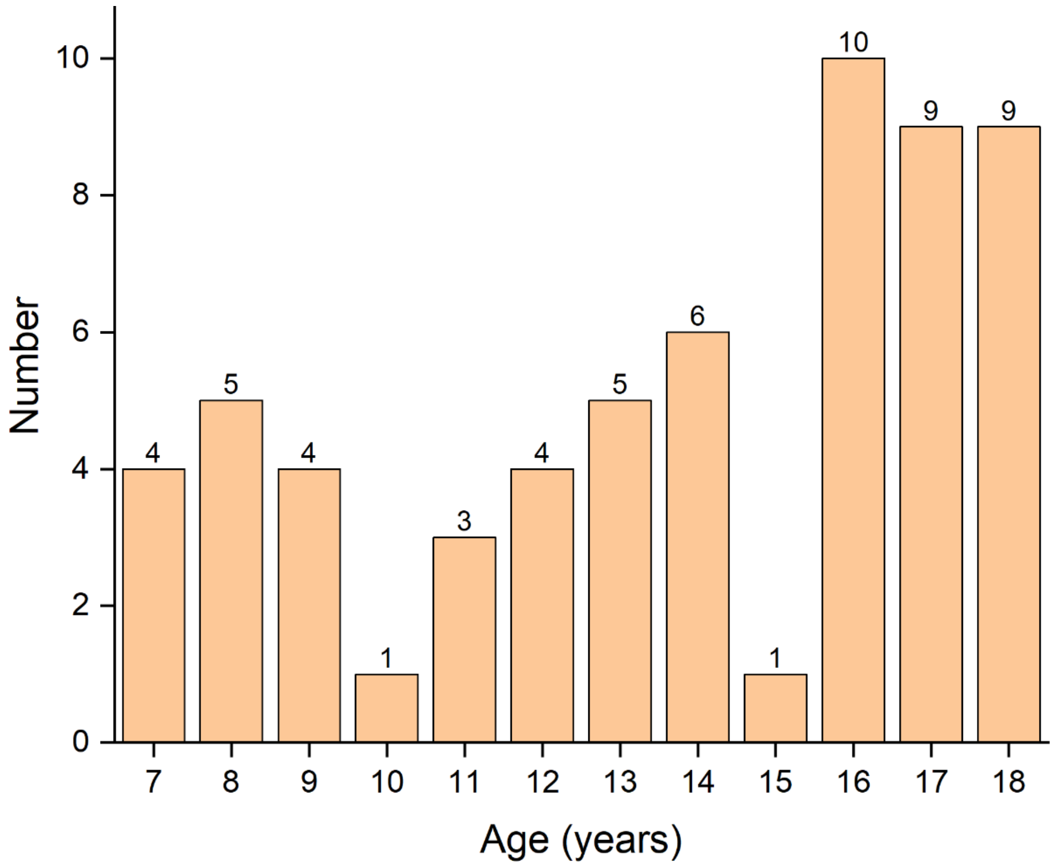

Figure S1 Age distribution of 61 pediatric patients with spinal ependymomas.
